# Prizes of the Paris Academy of Medicine, for 1862

**Published:** 1860-05

**Authors:** 


PRIZES OF THE PARIS ACADEMY OF MEDICINE FOR THE YEAR 1862.—The subjects for the Alhumbert prizes for the above year are—1st. "Endeavor, by well-conducted experiments, to throw new light upon the question of so-called spontaneous generation." A gold medal, of the value of five hundred dollars, will be awarded to the author of the best work upon this subject, published before the 1st of October, 1862. 2d. "Experimental study of the changes which may be caused in the development of the embryo of vertebrated animals by the action of external agents." The value of this prize is the same as that of the above, and the papers should be sent in before the 1st of April, 1862.

